# Supplementary material for: Prediction of Acute Respiratory Distress Syndrome in Traumatic Brain Injury Patients Based on Machine Learning Algorithms
Source: Medicina (Kaunas). 2023 Jan 15;59(1):171. doi: 10.3390/medicina59010171 (PMC9864532; doi:10.3390/medicina59010171)
Supplement: Supplementary file 1 [file medicina-59-00171-s001.zip › medicina-2071988-supplementary.pdf]

Supplementary Table S1. The comparison between TBI patients with complete data and those without records of PaO_2 and corresponding FiO_2.				
Variables	Overall (n=2280)	Group without PaO_2 and corresponding FiO_2 (n=1631, 71.5%)	Complete data group (n=649, 28.5%)	p
Age (year)	64.9 (43.7-81.0)	66.8 (45.9-82.7)	59.3 (38.8-77.4)	**<0.001**
Male gender, n (%)	1400 (61.4%)	976 (59.8%)	424 (65.3%)	**0.015**
Comorbidities				
Diabetes, n (%)	351 (15.4%)	258 (15.8%)	93 (14.3%)	0.374
Hypertension, n (%)	844 (37.0%)	657 (40.3%)	187 (28.8%)	**<0.001**
Hyperlipidemia, n (%)	298 (13.1%)	251 (15.4%)	47 (7.2%)	**<0.001**
Coronary heart disease, n (%)	293 (12.9%)	239 (14.7%)	54 (8.3%)	**<0.001**
Liver disease, n (%)	94 (4.1%)	73 (4.5%)	21 (3.2%)	0.179
Chronic renal disease, n (%)	153 (6.7%)	129 (7.90%)	24 (3.7%)	**<0.001**
Malignancy, n (%)	238 (10.4%)	196 (12.0%)	42 (6.5%)	**<0.001**
Vital signs on admission				
Systolic blood pressure (mmHg)	132 (117-147)	133 (119-148)	130 (113-146)	**<0.001**
Diastolic blood pressure (mmHg)	67 (56-77)	68 (58-78)	63 (53-74)	**<0.001**
Heart rate (s^{-1})	83 (72-96)	83 (72-96)	84 (71-97)	0.445
Respiratory rate (s^{-1})	18 (15-20)	18 (15-20)	17 (14-20)	**0.045**
GCS	12 (6-15)	14 (9-15)	6 (3-9)	**<0.001**
AIS chest	0 (0-0)	0 (0-0)	0 (0-3)	**<0.001**
ISS	16 (16-25)	16 (16-21)	20 (16-29)	**<0.001**
Laboratory tests				
WBC (10^9/L)	11.60 (8.40-15.70)	11.10 (8.10-14.70)	13.40 (10.00-18.10)	**<0.001**
Platelet (10^9/L)	230 (183-285)	232 (185-284)	228 (175-288)	0.341
RBC (10^9/L)	4.13 (3.67-4.57)	4.15 (3.71-4.60)	4.07 (3.60-4.51)	**0.004**
Hemoglobin (g/dL)	12.80 (11.40-14.10)	12.80 (11.40-14.20)	12.70 (11.20-14)	0.153
Glucose (mg/dL)	132 (110-165)	126 (107-154)	149 (121-186)	**<0.001**
Blood urea nitrogen (mg/dL)	16 (12-23)	17 (12-23)	16 (12-22)	**0.013**
Serum creatinine (mg/dL)	0.90 (0.70-1.10)	0.90 (0.80-1.10)	0.90 (0.70-1.10)	0.513
Serum sodium (mmol/L)	139 (137-141)	139 (137-141)	140 (137-142)	**0.025**
Serum potassium (mmol/L)	4.0 (3.7-4.4)	4.0 (3.7-4.4)	3.9 (3.6-4.3)	**<0.001**
Serum chloride (mmol/L)	104 (101-107)	104 (101-106)	105 (102-109)	**<0.001**
Serum calcium (mmol/L)	8.20 (1.14-8.90)	8.50 (7.20-9.00)	1.17 (1.06-8.20)	**<0.001**
Prothrombin time (s)	13.0 (12.3-14.2)	13.0 (12.2-14.1)	13.2 (12.6-14.3)	**<0.001**
INR	1.1 (1.0-1.3)	1.1 (1.0-1.2)	1.2 (1.1-1.3)	**<0.001**
Intracranial injury types				
Epidural hemorrhage, n (%)	543 (23.8%)	369 (22.6%)	174 (26.8%)	**0.034**
Subdural hemorrhage, n (%)	1319 (57.9%)	980 (60.1%)	339 (52.2%)	**<0.001**
Subarachnoid hemorrhage, n (%)	958 (42.0%)	662 (40.6%)	296 (45.6%)	**0.028**

Intraparenchymal hemorrhage, n (%)	447 (19.6%)	301 (18.5%)	146 (22.5%)	**0.028**
Treatments				
RBC during the first 24 hours, n (%)	178 (7.8%)	91 (5.6%)	87 (13.4%)	**<0.001**
Platelet during the first 24 hours, n (%)	223 (9.8%)	150 (9.2%)	73 (11.2%)	0.137
Anticoagulants during the first 24 hours, n (%)	569 (25.0%)	413 (25.3%)	156 (24.0%)	0.522
Antiplatelets during the first 24 hours, n (%)	63 (2.8%)	58 (3.6%)	5 (0.8%)	**<0.001**
Vasopressor during the first 24 hours, n (%)	150 (6.6%)	62 (3.8%)	88 (13.6%)	**<0.001**
Mechanical ventilation, n (%)	1104 (48.4%)	513 (31.5%)	591 (91.1%)	**<0.001**
Neurosurgery, n (%)	572 (25.1%)	313 (19.2%)	259 (39.9%)	**<0.001**
Length of ICU stay (days)	2.3 (1.2-5.6)	1.8 (1.1-3.5)	5.7 (2.4-12.1)	**<0.001**
Length of hospital stay (days)	6.3 (3.6-12.4)	5.5 (3.2-9.9)	10.1 (4.9-18.5)	**<0.001**
30-day mortality, n (%)	404 (17.7%)	213 (13.1%)	191 (29.4%)	**<0.001**

GCS, Glasgow Coma Scale; AIS, Abbreviated Injury Score; ISS, Injury Severity Score; WBC, white blood cell; RBC, red blood cell; INR, international normalized ratio; PaO_2, arterial oxygen pressure; FiO_2, fraction of inspired oxygen.
Bold values indicated p<0.05.
